# Systemic coagulation inflammation index and risk of mortality in peritoneal dialysis patients: a multicenter cohort study

**DOI:** 10.3389/fmed.2026.2005361

**Published:** 2026-10-26

**Authors:** Tianying Zhang, Xi Hua, Dong Sun, Xiangcheng Xie, Hong Hu, Wenkai Xia

**Affiliations:** 1 Department of Nephrology, Jiangyin People’s Hospital Affiliated to Nantong University, Jiangyin, China; 2 Department of Nephrology, Affiliated Hospital of Yangzhou University, Yangzhou First People’s Hospital, Yangzhou, China; 3 Department of Nephrology, The Affiliated Suzhou Hospital of Nanjing Medical University, Suzhou Municipal Hospital, Suzhou, China; 4 Department of Nephrology, Affiliated Hangzhou First People’s Hospital, Zhejiang University School of Medicine, Hangzhou, Zhejiang, China; 5 Nephrologisches Zentrum, Medizinische Klinik und Poliklinik IV, Klinikum der Universität München, Ludwig-Maximilians-University Munich, Munich, Germany

**Keywords:** biomarker, index, peritoneal dialysis, prognosis, systemic coagulation inflammation

## Abstract

**Background:**

The systemic coagulation inflammation (SCI) index has been investigated as a prognostic marker in several diseases. The aim of this study was to investigate the association of SCI with mortality in patients undergoing peritoneal dialysis (PD).

**Methods:**

A total of 324 incident PD patients who initiated treatment between January 2011 and December 2020 were retrospectively analyzed. Patients were stratified using the previously selected outcome-specific SCI cutoffs. Mortality associations were assessed using Cox regression and, for cardiovascular disease (CVD) mortality, competing-risk methods.

**Results:**

Patients were stratified with three groups based on the optimal cutoff value of SCI index. In the primary model (Model 2), lower SCI was associated with increased all-cause mortality (hazard ratio [HR], 9.32; 95% CI, 4.55–19.08, *P* < 0.001). For CVD mortality, the corresponding HR was 7.03 (95% CI, 3.83–12.92, *P* < 0.001). SCI areas under the receiver operating characteristic (ROC) curve were 0.681 and 0.688 for all-cause and CVD mortality, respectively. Significant differences in overall survival were observed in older and hypertension population.

**Conclusions:**

Lower SCI index was associated with higher mortality in this PD cohort. Discrimination was modest, and the data-derived cutoffs require independent validation before clinical use.

## Introduction

End-stage renal disease (ESRD) is severely life-threatening and characterized by irreversible loss of renal function, systemic inflammation, and other devastating complications that contribute to a substantial economic burden and public health concerns (1). Cardiovascular disease remains the leading cause of death among patients receiving peritoneal dialysis (PD), and PD-related as well as ESRD-related factors contribute to this excess risk (2). It is well recognized that the clinical deterioration of ESRD is closely correlated with systemic inflammatory response and coagulopathic disorders (3–5). Early detection and intervention of these risk factors are important for the clinical care of ESRD patients (6).

Accordingly, the systemic coagulation inflammation (SCI) index, a composite coagulation-inflammation indicator, has been investigated in patients undergoing acute type A aortic dissection surgery and in acute coronary syndrome (7, 8). In addition, recent studies reported that SCI index was associated with arteriovenous fistula failure and arteriovenous graft thrombosis in hemodialysis patients (9, 10). Inflammation- and nutrition-related composite indices, including the systemic immune-inflammation index and prognostic nutritional index, have also been associated with mortality in PD patients (11–13). Despite these findings, data on the association of the SCI index with prognosis in ESRD patients undergoing peritoneal dialysis (PD) are limited. The purpose of our study is to investigate the association of the SCI index with all-cause and cardiovascular disease (CVD) mortality in PD patients.

## Patients and methods

### Patients and data collection

We conducted a retrospective multi-center observational cohort study in patients with end-stage renal disease who initiated peritoneal dialysis (PD) between January 2011 and December 2020 at the Jiangyin People's Hospital Affiliated to Nantong University, Affiliated Hangzhou First People's Hospital, Zhejiang University School of Medicine, Affiliated Suzhou Hospital of Nanjing Medical University, Suzhou Municipal Hospital and Affiliated Hospital of Yangzhou University, Yangzhou First People's Hospital. Patients were excluded if they were younger than 18 years, received PD treatment for fewer than three consecutive months, had a history of hemodialysis or renal transplantation before PD, or lacked baseline SCI index or follow-up data. The reported follow-up period extended from PD initiation to death or administrative censoring on 31 December 2021. Baseline demographic, clinical, and laboratory data were collected within 3 months after PD treatment, and included age, sex, history of diabetes mellitus (DM), hypertension and cardiovascular disease (CVD), body mass index (BMI), systolic blood pressure, diastolic blood pressure, white blood cell (WBC), platelet, neutrophil, lymphocyte, serum albumin, globulin, creatinine, urea nitrogen, fibrinogen, uric acid, intact parathyroid hormone (iPTH), total cholesterol, triglyceride, calcium, phosphorus, potassium, total Kt/V and estimated glomerular filtration rate (eGFR). Informed consent was waived by the Ethics Committee due to the retrospective and non-interventional nature of the study.

### Study definition

The SCI index was calculated using the formula: SCI = platelet count (×10⁹/L) × fibrinogen (g/L)/WBC count (×10⁹/L). The systemic immune-inflammation index (SII) was calculated as SII = platelet count (×10⁹/L) × neutrophil count (×10⁹/L)/absolute lymphocyte count (×10⁹/L). The prognostic nutritional index (PNI) was calculated as albumin (g/L) + 5 × absolute lymphocyte count (×10⁹/L) (13). Lymphocyte percentage was not used. All three indices were recalculated from their components after cleaning and rounded to one decimal; full-precision SCI gave identical group assignments. The primary outcome was all-cause mortality, defined as death from any cause. The secondary outcome was CVD mortality, defined as death caused by cardiac arrhythmia, acute myocardial infarction, congestive heart failure, or peripheral vascular disease. Non-CVD deaths were identified as all-cause deaths without a CVD death indicator. Patients with a previous history of type 1 or 2 DM or who reported current use of oral glucose-lowering agents or insulin were considered to have DM. Hypertension was defined as current treatment with antihypertensive drugs or two separate blood pressure measurements ≥140/90 mmHg.

### Statistical analysis

Continuous variables are presented as mean ± standard deviation (SD), except uric acid and intact parathyroid hormone (iPTH), which are summarized as median [interquartile range (IQR)]. Categorical variables are presented as number (percentage), using the available observations as denominators. Three-group comparisons used one-way analysis of variance, Kruskal–Wallis tests, and Pearson chi-squared tests for categorical variables. These baseline comparisons were descriptive. Pearson correlations were calculated between SCI and laboratory measurements. Outcome-specific cutoffs originally selected using X-tile (14) were retained: <47.8, 47.8 to <118.7, and ≥118.7 for all-cause mortality, and <46.9, 46.9 to <74.9, and ≥74.9 for CVD mortality. Figure 1 documents the original X-tile selection (Yale University, New Haven, USA). Quartiles were calculated once from the available measurements in the author-corrected 319-patient extract, using linear interpolation. Values below Q1−3 × IQR or above Q3 + 3 × IQR were set to missing; other measurements from the same patient were retained. The thresholds were not recalculated after exclusion. The rule identified statistically extreme measurements and did not establish measurement error. Analyses before and after this *post hoc* cleaning were compared to assess sensitivity to the rule.

**Figure 1:**
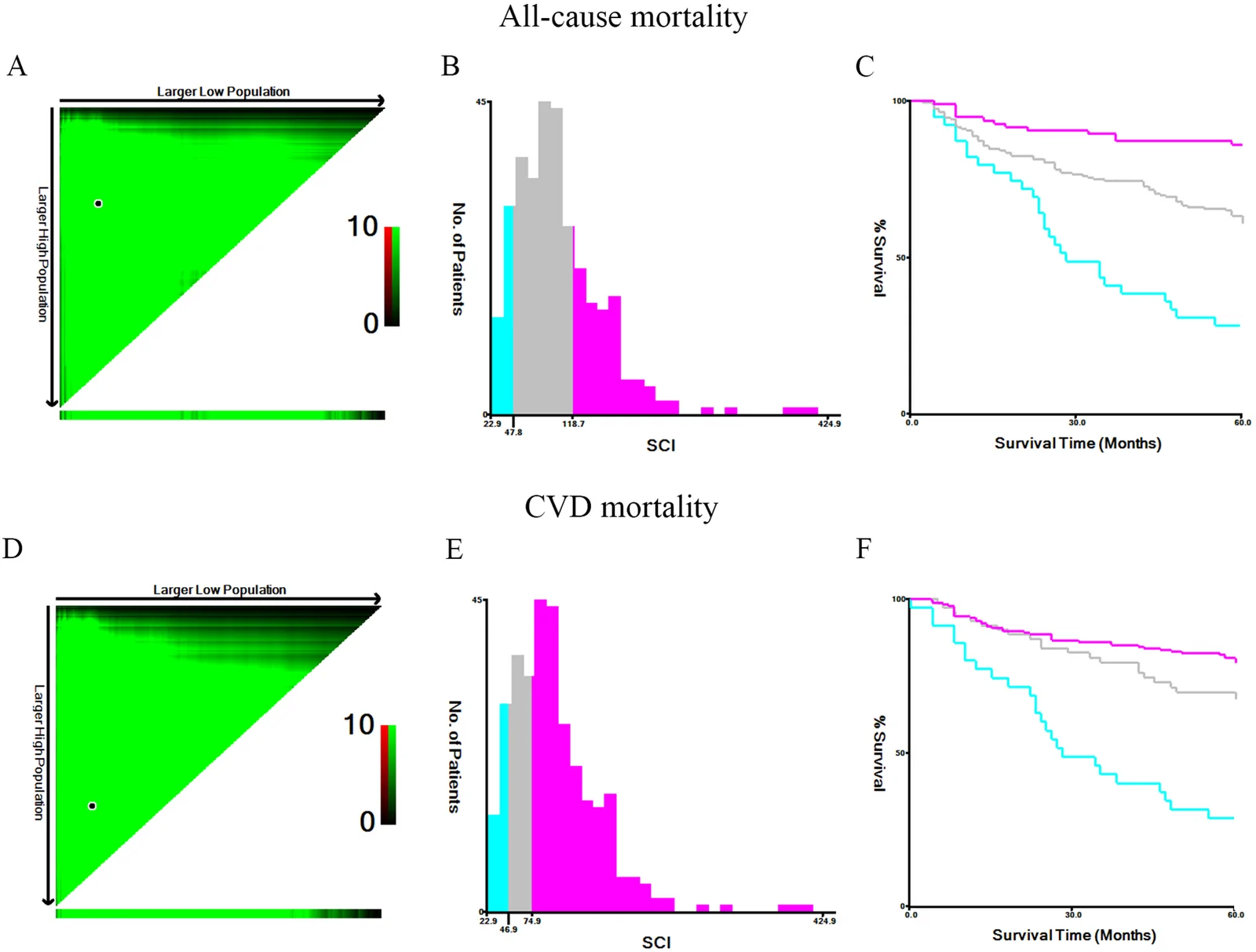
SCI cut-off thresholds determined by X-tile for all-cause and CVD mortality in patients undergoing PD. The original threshold-search plots are shown in **A** and **D**, SCI distributions in **B** and **E**, and corresponding Kaplan–Meier displays in **C** and **F**. The cutoffs were 47.8 and 118.7 for all-cause mortality and 46.9 and 74.9 for CVD mortality. CVD, cardiovascular disease; PD, peritoneal dialysis; SCI, systemic coagulation inflammation.

Kaplan–Meier curves and log-rank tests were used for all-cause survival. CVD cumulative incidence was estimated with non-CVD death as a competing event and compared using Gray's test. Cause-specific Cox models censored non-CVD deaths at death, whereas Fine–Gray models estimated subdistribution hazard ratios (sHRs). The high SCI category was the reference for each outcome. Covariates were entered simultaneously without stepwise selection or univariable *P*-value screening. Model 1 adjusted for age, sex, and BMI. Model 2 additionally adjusted for hypertension, DM, and prior CVD. Model 3 additionally included hemoglobin, albumin, globulin, total cholesterol, triglycerides, uric acid, iPTH, calcium, phosphorus, potassium, total Kt/V, and eGFR. Model 2 was emphasized to limit model complexity; Model 3 was treated as an expanded sensitivity analysis. For Cox models, proportional hazards were assessed using scaled Schoenfeld residual score tests with the Kaplan–Meier time transformation, including a global test. Variance inflation factors (VIFs) were calculated from each fitted design matrix. Tests were two-sided, with *P* < 0.05 used for nominal inference or multiplicity-adjusted *P* values where specified. Statistical analysis was performed using SPSS 23.0 software (SPSS Inc., IBM, USA).

## Results

### Patient characteristics

The source dataset contained 319 patients for the SCI analyses, including 192 men (60.2%). Mean age was 51.8 ± 16.2 years, and mean BMI was 22.7 ± 3.4 kg/m^2^. The low SCI group was younger and had lower prevalence of hypertension and DM, higher diastolic blood pressure and creatinine, and lower globulin and eGFR (Table 1).

**Table 1:** Baseline characteristics by all-cause mortality SCI category.

Variables	SCI			*P* value
	Low <47.8 (*n*=36)	Middle 47.8–<118.7 (*n*=188)	High ≥118.7 (*n*=95)	
Age (years)	44.4 ± 17.7	53.3 ± 15.8	51.7 ± 15.9	0.011
Male sex, *n* (%)	23 (63.9)	109 (58.0)	60 (63.2)	0.626
BMI (kg/m²)	21.8 ± 3.0	22.7 ± 3.5	23.0 ± 3.4	0.251
SBP (mmHg)	156.2 ± 26.4	151.1 ± 23.1	147.3 ± 25.8	0.157
DBP (mmHg)	96.6 ± 19.7	86.9 ± 15.6	85.7 ± 17.2	0.003
Hypertension, *n* (%)	19 (52.8)	131 (70.4)	76 (80.0)	0.008
DM, *n* (%)	1 (2.8)	47 (25.0)	29 (30.5)	0.004
Prior CVD, *n* (%)	2 (5.6)	23 (12.2)	10 (10.5)	0.509
Albumin (g/L)	34.8 ± 5.3	34.3 ± 4.4	33.2 ± 5.1	0.120
Globulin (g/L)	22.4 ± 4.5	24.9 ± 5.1	26.5 ± 5.2	<0.001
Hemoglobin (g/L)	90.0 ± 21.2	90.5 ± 19.2	88.2 ± 18.0	0.631
Creatinine (µmol/L)	967.9 ± 351.8	824.8 ± 326.2	718.6 ± 276.7	<0.001
BUN (mmol/L)	23.9 ± 11.2	22.0 ± 9.4	22.6 ± 9.1	0.514
Total cholesterol (mmol/L)	4.1 ± 1.2	4.4 ± 1.1	4.4 ± 1.2	0.302
Triglycerides (mmol/L)	1.6 ± 1.0	1.7 ± 1.0	1.8 ± 1.1	0.467
Uric acid (µmol/L)	460.0 (388.0–527.0)	439.2 (359.7–515.8)	429.1 (363.0–511.3)	0.579
iPTH (pmol/L)	123.1 (18.8–254.0)	70.4 (20.7–259.9)	168.9 (29.3–291.6)	0.190
Calcium (mmol/L)	2.2 ± 0.3	2.0 ± 0.3	2.0 ± 0.3	0.052
Phosphorus (mmol/L)	1.7 ± 0.5	1.8 ± 0.6	1.7 ± 0.5	0.494
Potassium (mmol/L)	3.9 ± 0.6	4.1 ± 0.8	4.2 ± 0.8	0.157
eGFR (mL/min/1.73 m²)	5.9 ± 3.1	6.4 ± 2.8	7.6 ± 2.9	0.001
Total Kt/V	2.2 ± 0.7	2.1 ± 0.6	2.1 ± 0.6	0.901

BMI, body mass index; SBP, systolic blood pressure; DBP, diastolic blood pressure; DM, diabetes mellitus; CVD, cardiovascular disease; BUN, blood urea nitrogen; iPTH, intact parathyroid hormone; eGFR, estimated glomerular filtration rate; SCI, systemic coagulation–inflammation index.

### Association of SCI index with clinical characteristics in PD patients

SCI index was positively correlated with globulin (r = 0.288, *P* < 0.001) and eGFR (r = 0.198, *P* < 0.001), and negatively correlated with creatinine (r = −0.226, *P* < 0.001; Table 2).

**Table 2:** Pearson correlations of SCI with laboratory measurements.

Variables	Globulin	Creatinine	eGFR
*r*	0.288	−0.226	0.198
*P* value	<0.001	<0.001	<0.001

eGFR, estimated glomerular filtration rate; SCI, systemic coagulation–inflammation index.

### Association of SCI index with patient outcomes

Among the 319 patients with available SCI, there were 115 deaths, comprising 87 CVD deaths and 28 non-CVD deaths. All-cause survival differed across SCI categories (log-rank *χ*^2^ = 41.612, *P* < 0.001). In Model 3, low versus high SCI was associated with an all-cause mortality HR of 13.71 (95% CI, 5.21–36.10), and middle versus high SCI with an HR of 4.27 (95% CI, 1.91–9.52) (both *P* < 0.001; Table 3). The global proportional hazards tests were not statistically significant in Models 1 and 2. In Model 3, global tests indicated departures from proportional hazards for all-cause mortality (*P* = 0.006) and CVD mortality (*P* = 0.009), respectively (Supplementary Table S1).

**Table 3:** Cox associations of SCI with mortality.

		Middle SCI		Low SCI	
Outcome/ Model	High SCI (reference)	HR (95% CI)	*P* value	HR (95% CI)	*P* value
All-cause mortality					
Crude	Ref	3.39 (1.88–6.11)	<0.001	7.54 (3.85–14.78)	<0.001
Model 1	Ref	2.90 (1.60–5.26)	<0.001	8.66 (4.30–17.44)	<0.001
Model 2	Ref	2.89 (1.59–5.26)	<0.001	9.32 (4.55–19.08)	<0.001
Model 3	Ref	4.27 (1.91–9.52)	<0.001	13.71 (5.21–36.10)	<0.001
CVD mortality					
Crude	Ref	1.80 (1.08–3.00)	0.023	4.77 (2.84–8.01)	<0.001
Model 1	Ref	1.87 (1.08–3.23)	0.025	6.09 (3.46–10.70)	<0.001
Model 2	Ref	1.96 (1.12–3.43)	0.019	7.03 (3.83–12.92)	<0.001
Model 3	Ref	2.10 (1.12–3.95)	0.021	7.88 (3.53–17.58)	<0.001

Model 1: age, sex, and BMI. Model 2: Model 1 plus hypertension, DM, and prior CVD. Model 3: Model 2 plus hemoglobin, albumin, globulin, total cholesterol, triglycerides, uric acid, iPTH, calcium, phosphorus, potassium, total Kt/V, and eGFR.

BMI, body mass index; SBP, systolic blood pressure; DBP, diastolic blood pressure; DM, diabetes mellitus; CVD, cardiovascular disease; BUN, blood urea nitrogen; iPTH, intact parathyroid hormone; eGFR, estimated glomerular filtration rate; SCI, systemic coagulation–inflammation index; HR, hazard ratio; CI, confidence interval.

CVD cumulative incidence differed across groups (Gray's *χ*^2^ = 36.387, *P* < 0.001). At 60 months, estimated CVD cumulative incidence was 68.8%, 33.3%, and 20.1% in the low, middle, and high categories, respectively. In Model 3, the cause-specific HRs were 7.88 (95% CI, 3.53–17.58) for low versus high SCI (*P* < 0.001) and 2.10 (95% CI, 1.12–3.95) for middle versus high SCI (*P* = 0.021). Corresponding Fine–Gray sHRs were 7.86 (95% CI, 3.48–17.75, *P* < 0.001) and 2.07 (95% CI, 1.12–3.84, *P* = 0.020) (Figure 2 and Supplementary Table S2). Subgroup analysis was performed to further evaluate the prognostic performance of SCI index in PD patients. We found patients with lower SCI index level had significantly higher all-cause mortality in patients older than 55 years and those with a history of hypertension (Table 4).

**Figure 2:**
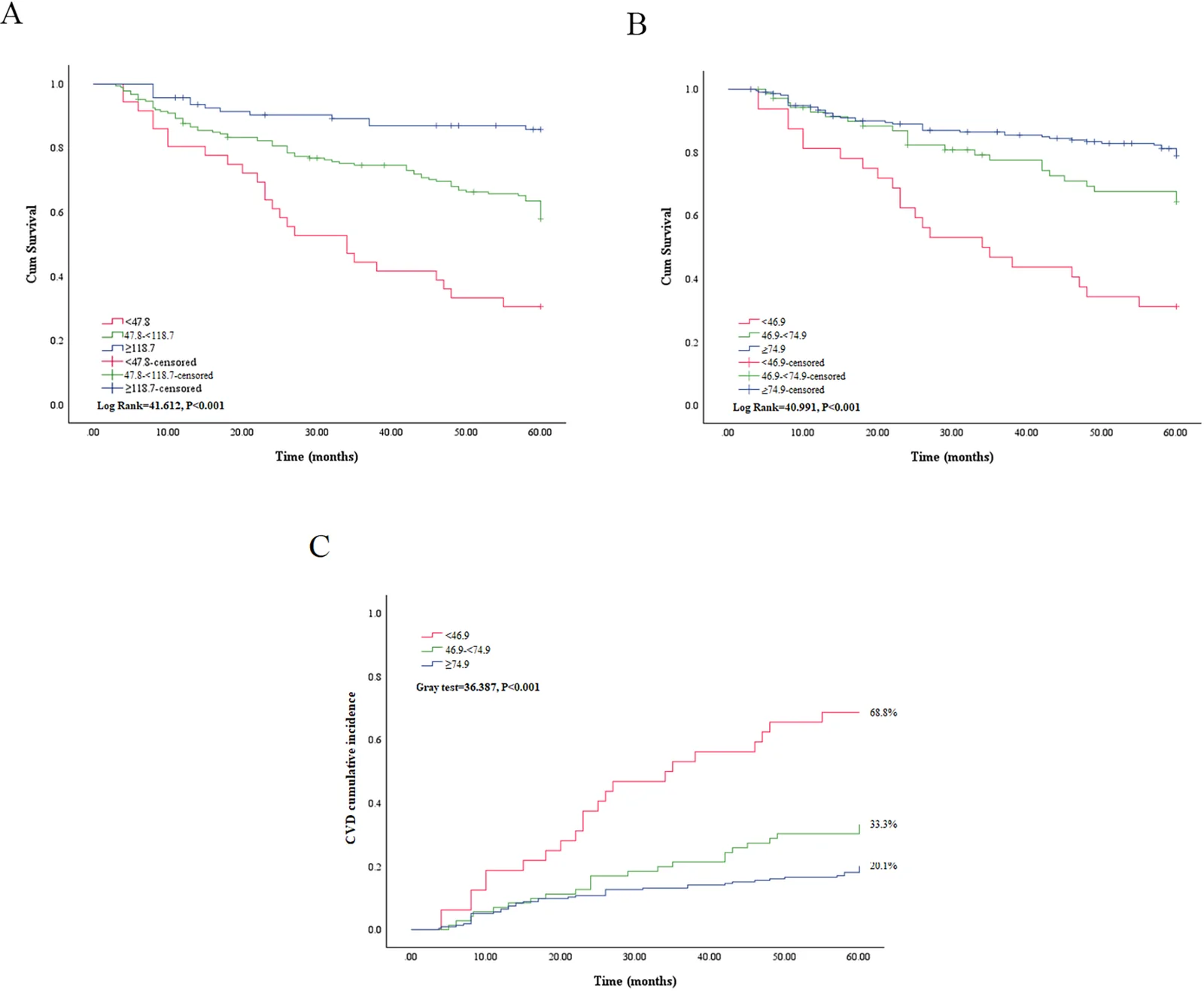
Survival **(A,B)** and CVD cumulative incidence **(C)** according to SCI category. CVD, cardiovascular disease; PD, peritoneal dialysis; SCI, systemic coagulation inflammation.

**Table 4:** Subgroup analysis of the predictive value of SCI index for overall survival.

Characteristics	HR (95% CI)	*P*	*P* for interaction
Age			0.021
<55	0.99 (0.74–1.32)	0.875	
≥55	1.55 (1.39–1.86)	<0.001	
Gender			0.207
Male	1.03 (0.64–2.09)	0.425	
Female	1.32 (0.42- 2.20)	0.631	
BMI			0.138
<23	0.98 (0.76–1.21)	0.209	
≥23	1.31 (0.71–2.53)	0.366	
Hypertension			0.020
No	0.33 (0.93–1.15)	0.081	
Yes	2.33 (1.14–4.74)	0.020	
DM			0.342
No	0.85 (0.36–2.03)	0.717	
Yes	1.03 (0.85–1.21)	0.843	
CVD			0.333
No	1.15 (0.86–2.09)	0.887	
Yes	1.26 (0.50–3.22)	0.627	
eGFR (mL/min/1.73 m²)			0.828
<6	1.28 (0.81–3.70)	<0.001	
≥6	1.33 (0.73–3.24)	<0.001	
Total Kt/V			0.054
<2.10	2.11 (1.77–5.08)	<0.001	
≥2.10	1.10 (0.46–3.82)	0.011	

SCI, systemic coagulation inflammation index; CVD, cardiovascular disease; BMI, body mass index; eGFR, estimated glomerular filtration rate; HR, hazard ratio; CI, confidence interval.

### The discrimination of SCI index for all-cause and CVD mortality

In the common sample of 319 patients with all six markers available, SCI showed modest discrimination for recorded all-cause mortality (AUC 0.681; 95% CI, 0.621–0.741, *P* < 0.001) and CVD mortality (AUC 0.688; 0.622–0.755, *P* < 0.001). For all-cause mortality, the comparator AUCs were 0.603 (95% CI, 0.538–0.667, *P* < 0.001) for platelet count, 0.599 (95% CI, 0.534–0.664, *P* < 0.001) for fibrinogen, 0.534 (95% CI, 0.469–0.599, *P* = 0.009) for WBC count, 0.503 (95% CI, 0.436–0.570, *P* = 0.907) for PNI, and 0.513 (95% CI, 0.447–0.579, *P* = 0.182) for SII. The corresponding CVD AUCs were 0.626 (95% CI, 0.557–0.696, *P* < 0.001), 0.590 (95% CI, 0.516–0.664, *P* < 0.001), 0.517 (95% CI, 0.446–0.587, *P* = 0.007), 0.548 (95% CI, 0.475–0.621, *P* = 0.985), and 0.515 (95% CI, 0.446–0.585, *P* = 0.289), respectively Figure 3.

**Figure 3:**
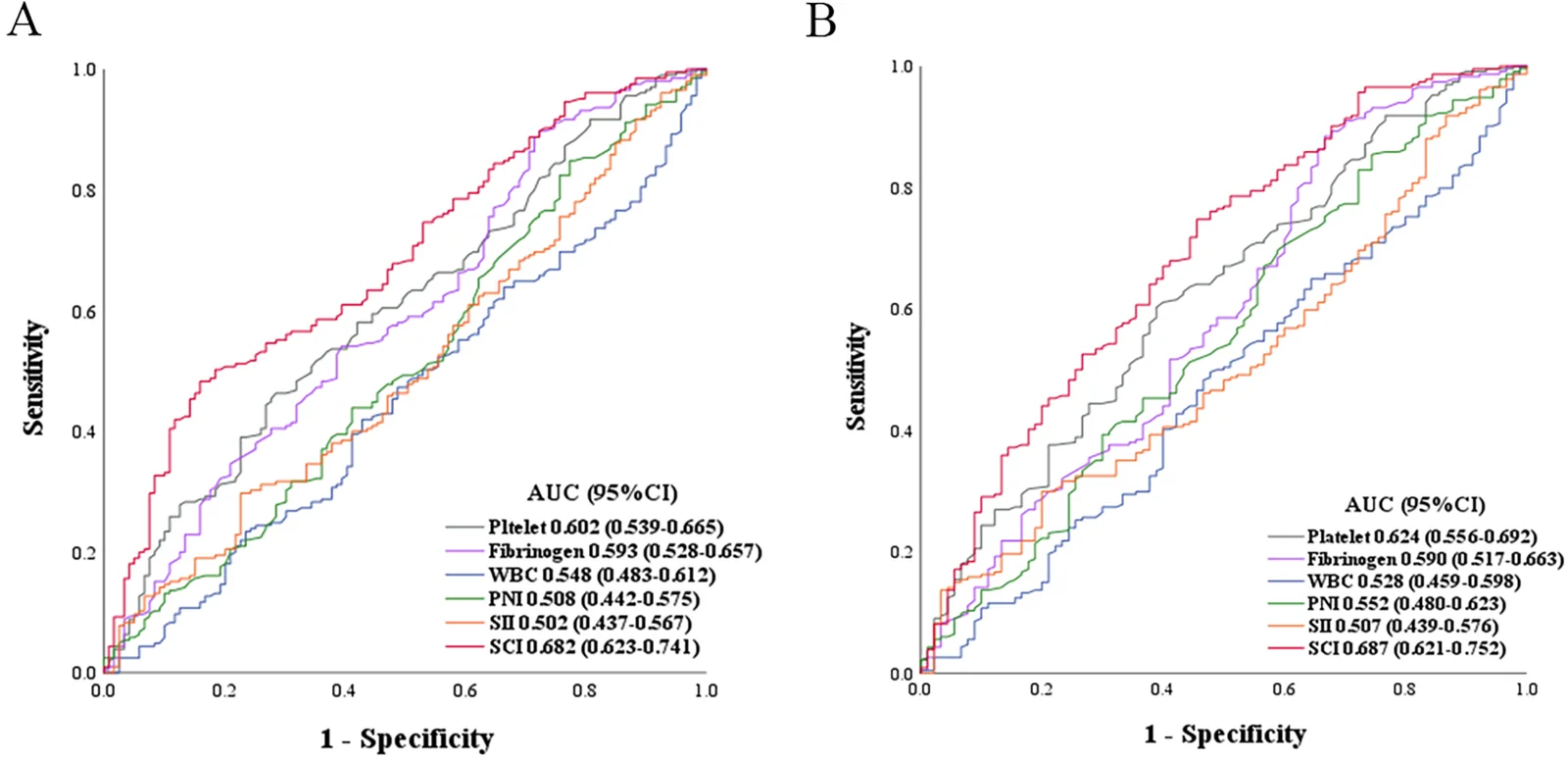
Receiver operating characteristic (ROC) curves for recorded all-cause **(A)** and CVD **(B)** mortality in patients undergoing PD. AUC, area under the ROC curve; CI, confidence interval; CVD, cardiovascular disease; PD, peritoneal dialysis; PNI, prognostic nutritional index; SCI, systemic coagulation inflammation; SII, systemic immune-inflammation index; WBC, white blood cell.

## Discussion

In this multicenter retrospective cohort of incident peritoneal dialysis (PD) patients, the systemic coagulation-inflammation (SCI) index was independently associated with both all-cause and cardiovascular mortality. Patients with a lower SCI index had a markedly higher risk of all-cause mortality and cardiovascular mortality after multivariable adjustment. After multiplicity correction, SCI had a higher AUC than platelet count, white blood cell count, PNI, and SII for all-cause mortality, and than white blood cell count and SII for CVD mortality. The comparisons did not support superiority over every marker. These findings suggest that SCI may reflect a combination of coagulation and inflammatory processes relevant to prognosis in PD; they do not establish a validated clinical prediction tool.

As reported previously, the SCI index has been investigated as a hematological indicator in various diseases (7, 15, 16). In acute type A aortic dissection surgery, lower SCI levels were associated with poorer short-term survival (8). Acute aortic dissection and chronic kidney disease differs in their pathophysiology, so an association in the same direction does not establish a shared mechanism. In acute aortic dissection, a low SCI index may reflect marked leukocytosis, platelet consumption, and fibrinogen depletion, which together indicate severe systemic inflammation and coagulopathy. In chronic kidney disease (CKD), low SCI might reflect inflammatory leukocyte elevation, impaired coagulation reserve, platelet abnormalities, or malnutrition-inflammation. These processes may be particularly prevalent in PD patients, in whom inflammation and hemostatic disturbance often coexist and are associated with cardiovascular events, infection susceptibility, and frailty (3, 17). The biological plausibility of this association is supported by the close interaction between inflammation, coagulation, vascular injury, malnutrition, and adverse outcomes in CKD (18, 19). CKD is characterized by persistent immune activation, endothelial dysfunction, oxidative stress, and metabolic dysregulation, all of which can contribute to cardiovascular injury and progressive systemic vulnerability (5, 18). Coagulation abnormalities are also common in CKD; prior observational evidence has shown that patients with nondialysis CKD exhibit higher levels of fibrinogen, von Willebrand factor, factor VII, factor VIII, and D-dimer than healthy controls, indicating a prothrombotic and endothelial-activated milieu (4). Because the SCI index is calculated from platelet count, fibrinogen, and white blood cell count, it may provide a simple composite measure of the balance between thrombo-inflammatory activation and hematologic reserve in dialysis patients. This mechanistic interpretation was not directly tested in the present study.

The higher numerical AUC of SCI in our cohort may reflect the integration of information from leukocyte counts, platelet counts, and fibrinogen. Elevated WBC counts have been associated with all-cause and cardiac mortality in PD, supporting the relevance of the inflammatory component (20). Combining WBC with platelet count and fibrinogen may capture variation across inflammatory and hemostatic processes that is less apparent from individual measurements. Compared with SII, SCI includes fibrinogen, which participates in coagulation and the acute-phase response (21). PNI captures a different aspect of risk through albumin and lymphocyte count; in PD, albumin is influenced by both inflammation and peritoneal protein loss (22). These differences provide plausible explanations for the observed discrimination, although they do not establish a biological mechanism. Higher fibrinogen alone has also been associated with mortality in PD (21), low SCI should therefore be interpreted in relation to its individual components. Moreover, the absence of significant differences in some pairwise AUC comparisons limits claim of superiority over all comparators.

Reevaluation in PD is warranted because reported associations of SCI with outcomes have differed across clinical settings: low SCI was associated with postoperative mortality in acute aortic dissection (8), whereas high SCI was associated with early arteriovenous graft thrombosis in hemodialysis (9). Our findings extend its evaluation to mortality in PD, where its potential clinical role is as an adjunct to existing risk assessment. If externally validated, a low SCI could help identify patients who warrant closer review of blood count abnormalities, inflammatory conditions, and nutritional status. Such use would complement the assessment of volume status, residual kidney function, and other clinical factors emphasized in goal-directed PD care (23). However, this study examined baseline SCI and subsequent mortality; it did not evaluate whether interventions intended to alter SCI improve survival or whether an SCI-guided care strategy benefits patients. The observed association does not justify attempting to increase SCI or selecting antithrombotic treatment on this basis. Prospective studies should establish the value of repeated measurements and incremental prediction beyond established clinical variables, followed by trials assessing whether SCI-guided management improves patient outcomes.

Several limitations affect interpretation. The retrospective design and absence of center identifiers in the analytic extract leave residual confounding and between-center variation unaddressed. SCI was measured once within three months of PD initiation and may vary with infection, nutritional status, liver function, medication, and intercurrent illness. Missing-data mechanisms were not established, and complete-case analyses may introduce selection bias. Although it was applied uniformly without reference to outcomes, it can exclude valid extreme measurements and changes both the analysis population and covariate distributions. The proportional subdistribution hazards assumption for the Fine–Gray models was not formally assessed. Ordinary ROC analyses did not adjust for censoring and do not establish discrimination at a fixed prediction horizon. Subgroup estimates were imprecise, including a stratum with no deaths in the high SCI reference group. The hypertension and age interaction were sensitive to cleaning and test choice and was based on one low-SCI patient. It should not guide subgroup-specific management. Calibration, incremental value beyond clinical predictors, and external validity remain to be assessed.

## Conclusion

In conclusion, our study demonstrated an association between lower SCI index and higher all-cause and CVD mortality in PD patients. SCI may have value for risk stratification, but independent validation and evidence that SCI-guided management improves outcomes are required before clinical use.

## Data Availability

The raw data supporting the conclusions of this article will be made available by the authors, without undue reservation.
