# Supplementary material for: Systemic coagulation inflammation index and risk of mortality in peritoneal dialysis patients: a multicenter cohort study
Source: Front Med (Lausanne). 2026 Oct 26;13:2005361. doi: 10.3389/fmed.2026.2005361 (PMC13745352; doi:10.3389/fmed.2026.2005361)
Supplement: Supplementary file 1 [file Table2.docx]

**Table S1. Proportional hazards and collinearity diagnostics for adjusted Cox models**

| **Outcome** | **Model** | **PH P Middle vs high** | **PH P Low vs high** | **Global PH P** | **VIF range** |
| --- | --- | --- | --- | --- | --- |
| All-cause | Model 1 | 0.417 | 0.608 | 0.431 | 1.039–1.248 |
| All-cause | Model 2 | 0.379 | 0.635 | 0.254 | 1.049–1.315 |
| All-cause | Model 3 | 0.328 | 0.617 | 0.006 | 1.266–1.957 |
| CVD | Model 1 | 0.451 | 0.341 | 0.455 | 1.049–1.100 |
| CVD | Model 2 | 0.468 | 0.361 | 0.190 | 1.064–1.324 |
| CVD | Model 3 | 0.159 | 0.393 | 0.009 | 1.148–1.952 |

Model 1 adjusts for age, sex, and BMI. Model 2 additionally adjusts for hypertension, diabetes, and prior CVD. Model 3 additionally includes hemoglobin, albumin, globulin, total cholesterol, triglycerides, uric acid, iPTH, calcium, phosphorus, potassium, total Kt/V, and eGFR.

**Table S2. Competing risk regression for CVD mortality**

| **Model** | **Middle SCI versus high SCI** | | **Low SCI versus high SCI** | |
| --- | --- | --- | --- | --- |
|  | **sHR (95% CI)** | **P value** | **sHR (95% CI)** | **P value** |
| Crude | 1.76 (1.06–2.91) | 0.028 | 4.95 (2.98–8.22) | <0.001 |
| Model 1 | 1.86 (1.08–3.19) | 0.025 | 6.30 (3.56–11.14) | <0.001 |
| Model 2 | 1.92 (1.10–3.35) | 0.023 | 7.21 (3.94–13.20) | <0.001 |
| Model 3 | 2.07 (1.12–3.84) | 0.020 | 7.86 (3.48–17.75) | <0.001 |

Crude models included SCI category only. Model 1 adjusted for age, sex, and BMI. Model 2 additionally adjusted for hypertension, diabetes mellitus, and prior CVD. Model 3 additionally adjusted for hemoglobin, albumin, globulin, total cholesterol, triglycerides, uric acid, intact parathyroid hormone, calcium, phosphorus, potassium, total Kt/V, and eGFR.

Abbreviations: SCI, systemic coagulation inflammation index; CVD, cardiovascular disease; HR, hazard ratio; sHR, subdistribution hazard ratio; CI, confidence interval; BMI, body mass index; eGFR, estimated glomerular filtration rate.
